# Comparative analysis of respiratory motion tracking using Microsoft Kinect v2 sensor

**DOI:** 10.1002/acm2.12318

**Published:** 2018-03-25

**Authors:** Evan Silverstein, Michael Snyder

**Affiliations:** ^1^ School of Medicine Wayne State University Detroit MI USA

**Keywords:** computer software, kinect, motion management, patient movement, respiratory motion

## Abstract

**Purpose:**

To present and evaluate a straightforward implementation of a marker‐less, respiratory motion‐tracking process utilizing Kinect v2 camera as a gating tool during 4DCT or during radiotherapy treatments.

**Methods:**

Utilizing the depth sensor on the Kinect as well as author written C# code, respiratory motion of a subject was tracked by recording depth values obtained at user selected points on the subject, with each point representing one pixel on the depth image. As a patient breathes, specific anatomical points on the chest/abdomen will move slightly within the depth image across pixels. By tracking how depth values change for a specific pixel, instead of how the anatomical point moves throughout the image, a respiratory trace can be obtained based on changing depth values of the selected pixel. Tracking these values was implemented via marker‐less setup. Varian's RPM system and the Anzai belt system were used in tandem with the Kinect to compare respiratory traces obtained by each using two different subjects.

**Results:**

Analysis of the depth information from the Kinect for purposes of phase‐ and amplitude‐based binning correlated well with the RPM and Anzai systems. Interquartile Range (IQR) values were obtained comparing times correlated with specific amplitude and phase percentages against each product. The IQR time spans indicated the Kinect would measure specific percentage values within 0.077 s for Subject 1 and 0.164 s for Subject 2 when compared to values obtained with RPM or Anzai. For 4DCT scans, these times correlate to less than 1 mm of couch movement and would create an offset of 1/2 an acquired slice.

**Conclusion:**

By tracking depth values of user selected pixels within the depth image, rather than tracking specific anatomical locations, respiratory motion can be tracked and visualized utilizing the Kinect with results comparable to that of the Varian RPM and Anzai belt.

## 1 INTRODUCTION

As radiotherapy treatments become increasingly precise, identifying and visualizing tumor movement during treatment becomes exceedingly important. Tumors located within the thorax and abdomen are significantly affected by motion induced with a patient's natural respiratory cycle. Accounting for this additional internal motion becomes paramount. One specific way to acquire and process this information is through the use of a 4DCT, by which the respiratory motion of the patient is tracked using a gating device.^1^, ^2^ The respiratory motion trace is processed in tandem with the CT acquisition and CT slices are binned to specific portions of the respiratory cycle.^3^ This process then allows internal motion visualization of the tumor by use of external motion tracking.^4^

Devices used to acquire the respiratory motion trace typically require some manner of physical device attached to the patient by way of a marker placed on the patient's surface or apparatus worn by the patient. However, these processes may require repositioning and multiple attempts to get an accurate respiratory motion trace due to irregular breathing and can restrict the respiratory motion tracking to one specific area on the patient, typically the lower abdomen. In this manuscript, the Microsoft Kinect v2 sensor was adapted to trace and record a patient's breathing cycle by way of a marker‐less process, doing away with any requirement for external hardware to be attached to the patient.

Developed and released by Microsoft in 2014, the Kinect v2 was created for the purposes of anatomical motion tracking by combining a high resolution color camera and a time‐of‐flight IR projector/sensor. Additionally, Microsoft released a software development kit (SDK) which is available free of charge. This kit contains sample programs which can facilitate access to various functions of the Kinect to software developers.^5^, ^6^ Allowing this open‐sourced platform has enabled developers to create a vast number of applications within the medical community ranging from tracking and management of inter‐ and intra‐fraction patient motion to gesture recognition within surgery suites for a hands‐free computer interface.^7^, ^8^ The combination of a color camera with an IR projector/sensor to obtain depth information has allowed the Kinect to become a versatile and useful tool within the medical community.

Previous research into respiratory motion tracking using the Kinect utilized either the Kinect v1 or required a translational marker to be placed on the patient's surface or embedded within clothing worn by the patient,^9^, ^10^, ^11^ similar to other respiratory tracking systems currently available for purchase. The latest version of the Kinect contains higher resolution sensors than the previous model, which helps remove the requirement for a translational marker to track respiratory motion. The removal of this requirement allows for a simpler process to be employed with less trial‐and‐error to obtain a useful respiratory trace.

In this manuscript, the Microsoft Kinect v2 sensor was adapted to trace and record a patient's breathing cycle by way of a marker‐less process. The cost of utilizing a marker‐less approach is the inability to guarantee tracking of a specific point on the patient's surface. This is due to the fact that the tracking process is done with respect to pixels in an image frame as opposed to fixed anatomical locations. Motion of the patient's surface during breathing will, in general, cause slightly different anatomical points within some connected surface area to pass through the tracked pixels within the image. This inherent difference between marker‐based and marker‐less tracking could theoretically lead to differences in recorded breathing traces between the methodologies. As a result, our evaluation of the Kinect v2 sensor as a motion‐tracking device also includes, by necessity, an overarching evaluation of a general marker‐less approach whereby the motion tracking is in some sense decoupled from the motion of singular points on the patient's surface.

## 2 MATERIALS AND METHODS

In this study, a Kinect respiratory tracking process was created and compared against both the Varian RPM Respiratory Gating system (RPM) and the Anzai Gating system (Anzai). For comparison and accuracy measurements, RPM and Anzai were both employed to a subject at the same time with the Kinect mounted above the patient. RPM traces the movement of a propriety marker placed on the subject's abdomen through the use of infrared sensors at a rate of 30 fps.^12^ Anzai utilizes a belt strapped around the subject's abdomen which contains a pressure sensor to track the respiratory motion at a rate of 40 fps.^13^, ^14^ The Kinect returns depth values, in mm, for every pixel within the depth frame at a rate of 30 fps.^15^ All three products acquired data simultaneously with the RPM marker placed directly on top of the Anzai belt and data was exported from all three for analysis.

Currently available gating procedures employ either a phase based or amplitude based binning process when incorporating respiratory motion.^16^, ^17^ As such, the traces recorded for all three products in this manuscript were analyzed with each process in mind. With a phase based binning process, the period of one cycle is obtained and divided up into ten phase portions with bins of equal width. With an amplitude based binning process, the bins are divided up into percentages of the maximum and minimum amplitude throughout one cycle, typically calculated as 100%, 80%, 60%, 40%, 20%, and 0%. These percentages correspond to specific physical states of the breathing cycle (mid‐inhalation, maximum exhalation, etc.). Given irregularities that can occur in a patient's breathing pattern which may cause shifts in the phase but not amplitude, many binning procedures are moving away from a phased based process in favor of an amplitude based process.^18^ However, in this manuscript, both binning procedures are used to test the validity of data being recorded by the Kinect.

Calculation and identification of the local maximum and minimum for each breathing cycle (100% amplitude, and 0% amplitude, respectively) was implemented through a simple local comparison algorithm. To mitigate possible misidentification of per‐cycle maxima and minimum due to temporally small, noisy perturbations, each individual data point of the trace was compared to the 10 data points acquired before and after, allowing for 20 comparisons in total. If the data point in question was greater than or equal to the 20 points surrounding it in time, it was considered 100% amplitude for that breathing cycle. If the data point was less than or equal to the 20 points surrounding it in time, it was considered 0% amplitude. Similar to analyses performed in the clinic when acquiring respiratory traces, multiple values of 100% or 0% amplitude may be identified by the system for the same breathing cycle. As such, manual adjustment was required to remove duplicate local maximum or minimums.

In order to obtain data for the respiratory trace, the Kinect v2's depth camera was utilized. The depth camera has a resolution of 512 × 424 and has the ability to detect distances ranging from 0.5 m to 4.5 m.^19^ The sensor returns depth data for each pixel within the 512 × 424 frame in 1 mm increments. Rather than track movement associated with a specific location on the body and monitor depth changes as it moves across the frame, as would be done with a physical marker, the system is designed to track specific pixels from the depth image and record the depth values returned over time. Although different from the typical respiratory tracking processes, which track a specific location on the body, this manuscript investigates if both processes can produce the same respiratory trace with congruent results.

To begin the data collection process, the user manually selects 5–12 points anywhere on the patient for respiratory motion tracking. Data collection duration is also selected by the user and the process can be stopped manually if needed. Each point has depth data continuously recorded during the acquisition process, with visual displays of each trace, and the program can choose the most accurate representation of the respiratory motion by calculating the largest difference between the maximum and minimum distances recorded for the points created. Additionally, as all traces during acquisition are saved, the user has the ability to view and select traces from different points to those chosen by the program in order represent respiratory motion if so desired.

To ensure that the data collection process and GUI were as user friendly as possible, the body tracking capabilities of the Kinect were implemented. The Kinect software has the ability to detect when a human body has entered the frame of the camera and can differentiate between pixels associated with a body vs pixels belonging to the background. Once the body is recognized by the Kinect, the background can be removed from the image displayed allowing for an easy visualization of the patient. The advantage to utilizing this process is that the image displayed is aligned, pixel for pixel, exactly to the depth images generated. This allows selection of specific points on the patient to exact depth data generated by the depth sensor.

In order to reduce noise as much as possible from the depth values obtained for each pixel selected, a median filtration algorithm was implemented for data obtained within a specific frame. Yang et al. measured typical noise from the depth sensor to be less than 2 mm when the object was within a 1–2 m range from the Kinect.^20^ Additionally, random fluctuations can act to produce a depth value of 0 or a value much greater than an expected depth. The median filtration algorithm implemented reduces this noise by creating a 7 × 7 grid of pixels around the pixel selected. Depth values from all 49 pixels are analyzed and the median of those pixels is used as the corrected value for the center pixel. This process enables noise filtration of the depth data without being affected by any outliers within the 7 × 7 grid.

During each tracking session, the Kinect was mounted directly over the subject pointing down at an angle of roughly 45 degrees and was set at a height of roughly 0.75 m. Data acquisitions were performed on both a male and female subject for approximately 120 s and each were asked to breath in a manner typical for the individual with no breath holds. Lachat et al. noted that the accuracy and constancy of depth values obtained from the Kinect requires a brief warm up period of approximately 30 min.^21^ As such, the Kinect was allowed ample warm up time during setup and before data was acquired.

## 3 RESULTS

**Figure 1:**
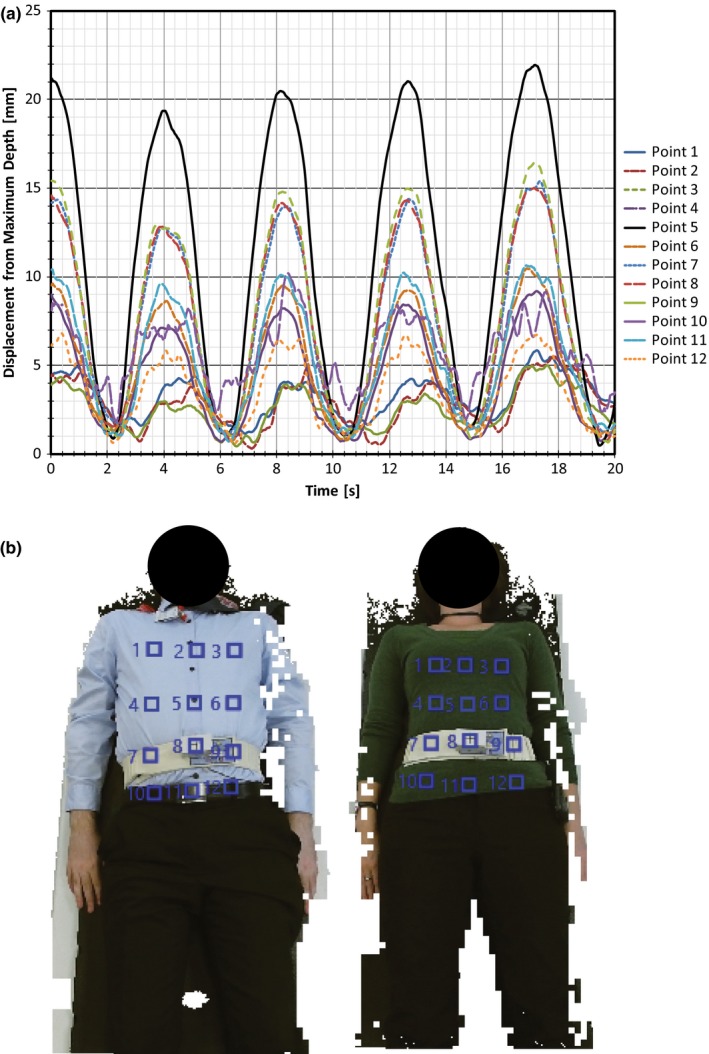
(a) Sample respiratory trace from Kinect with all 12 points selected by the user. Values along the *y*‐axis indicate the difference between the current depth value and the maximum depth value for that user selected point throughout the recorded trace. (b) Subjects 1 and 2 with points 1–12 selected.

Figure 1 displays a sample respiratory trace from the Kinect with all 12 points selected by the user as well as images of each subject with all 12 points shown. As previously mentioned, the point selected by the system to represent the respiratory motion is done so by calculating the largest amplitude between the traces created for all points selected. In this example, point 5 (located on the diaphragm) has the largest difference between the maximum and minimum values throughout the trace and, as such, it would be chosen by the system as the representation of the patient's respiratory motion. For analysis of the trace generated by the Kinect, point 5 from each subject was chosen to represent the respiratory motion. This allowed for analysis and comparison of a trace obtained from a location that was different from those obtained from RPM and Anzai while still containing amplitudes large enough to be compared to both systems.

Initial comparisons of the traces between systems involved implementing typical amplitude and phase based binning methods that would be used for gating purposes within the clinic. With the amplitude binning method, the maximum and minimum displacement values were obtained for each breathing cycle and amplitude values were obtained for 100%, 80%, 60%, 40%, 20%, and 0% of the local maximum value. The times at which each percentage occurred within each breathing cycle were then obtained across all three products. For the phase based binning method, the maximum displacement value was again utilized for each breathing cycle and the period of the cycle was divided into ten equal bins. The times for each bin were then obtained across all three products.

**Figure 2:**
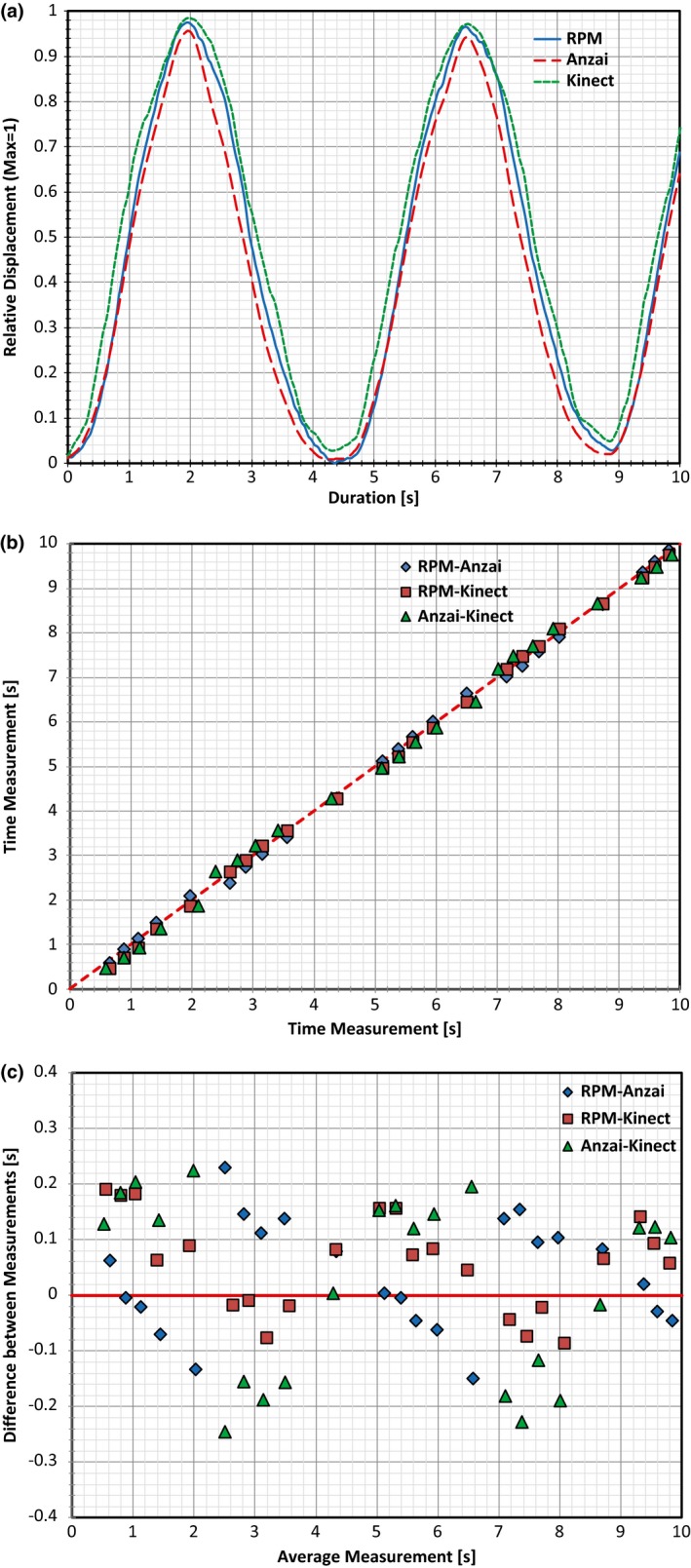
Plots created for Subject 1 based on two full respiratory cycles. (a) Plot with all three traces overlapped, (b) plot of time measurement vs time measurement obtained for the amplitude binning process for all three products comparisons, (c) Bland–Altman plot generated using the same measured values plotted in (b).

**Figure 3:**
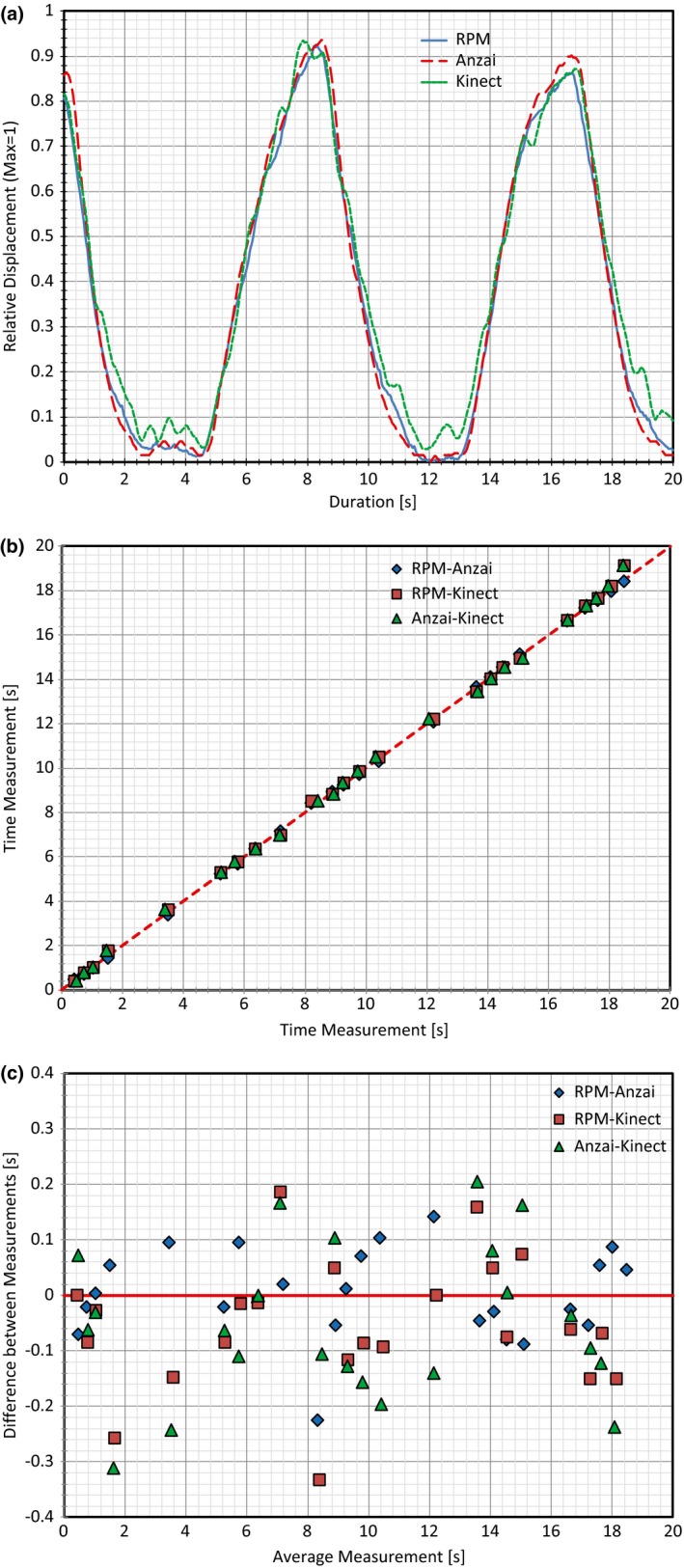
Plots created for Subject 2 based on two full respiratory cycles. (a) Plot with all three traces overlapped, (b) plot of time measurement vs time measurement obtained for the amplitude binning process for all three products comparisons, (c) Bland–Altman plot generated using the same measured values plotted in (b).

Portions of the data obtained with all three respiratory systems collecting data are displayed in Figs. 2(a) and 3(a). To align and overlap the data, the relative displacement was used based on the global maximum displacement during the respiratory tracking. Initial analysis of the times obtained for the amplitude binning process was accomplished using a Bland–Altman approach.^22^ First, measurements between two of the products were plotted along a line of Y = X for simple comparability [see Figs. 2(b) and 3(b) pertaining to Subjects 1 and 2, respectively] with one product measurement as the X coordinate for a point, and another product measurement as the Y coordinate. The closer each point is to the line of Y = X, the more similar the measurements. Next, all comparisons were analyzed utilizing a Bland–Altman plot to test for agreement as shown in Figs. 2(c) and 3(c) for Subjects 1 and 2, respectively. Here, the plot contains data comparing two products with each point on the plot having the X and Y coordinates calculated by the following:

(1) $$\left(X,Y\right)=\left(\frac{t_{A}+t_{B}}{2},t_{A}-t_{B},\right)$$

The X coordinate of a point, $\frac{t_{A}+t_{B}}{2}$, represents the average time measurement for a specific amplitude percentage between two products (t_A_ for product A, and t_B_ for product B). The Y value, $t_{A}-t_{B}$, represents the difference between the time measurements from the two products being compared. In essence, the difference between two time measurements for a specific amplitude percentage (Y value) is plotted against the average of those same two measurements (X value).^22^, ^23^ The data analyzed here with the Bland–Altman approach only represents the data obtained from the amplitude binning process. This was simply done for clarity as analysis for the phase based binning process would yields similar results.

**Figure 4:**
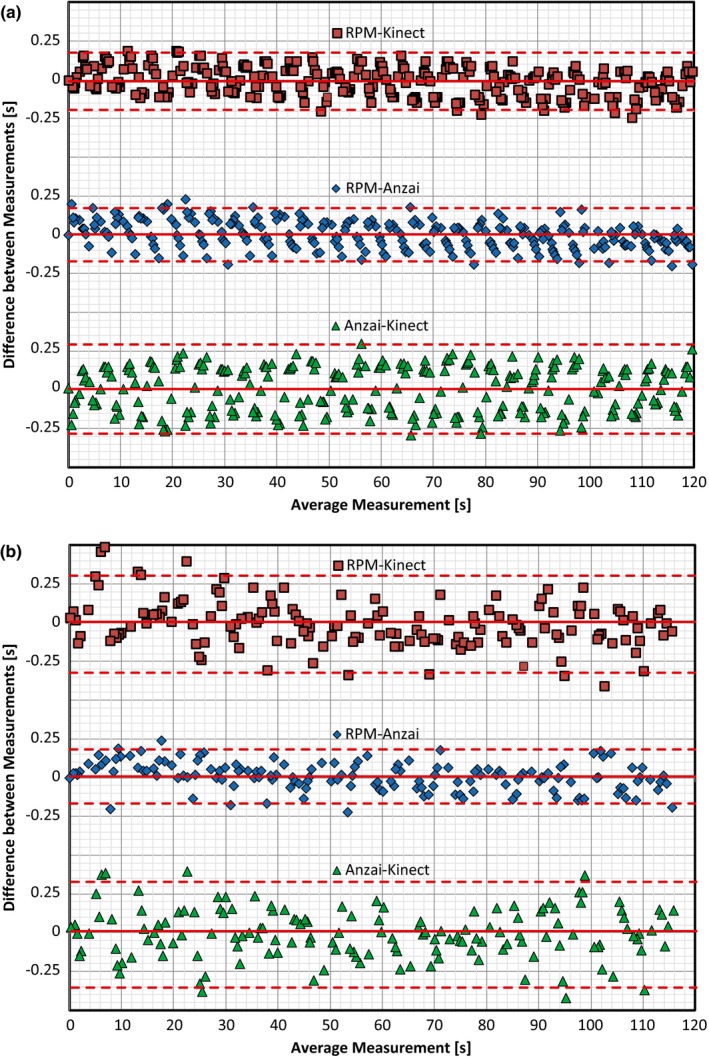
Bland–Altman plots generated for (a) Subject 1 and (b) Subject 2 based on time values obtained through the amplitude binning process. Comparisons were made between RPM and Kinect (top plot), RPM and Anzai (middle plot), and Anzai and Kinect (bottom plot). The solid line is the mean of all values and the dashed lines represent the mean ± 1.96 SD (i.e., the 95% confidence interval).

Further analysis utilized the Bland–Altman plots for amplitude time values obtained throughout the 120 s of recording. Here, the data is plotted around the line representing the mean for all measurements as well as lines representing the mean ± 1.96 × SD (i.e., the 95% Confidence Interval). Figure 4 displays comparisons from all three products with values obtained for Subject 1 and Subject 2.

With the Bland–Altman plots created in Fig. 4, the agreement between two products producing similar measurements lies with the percentage of values that fall within the span of the mean ± 1.96 × SD. Typically, two products can be shown to produce similar measurements if roughly 95% of the data within the plot falls inside this range. Table 1 summarizes the percent of values within the range specified and indicates that all three products have similar agreement with one another regarding the time values obtained for the amplitude percentages.

**Table 1:** Summary of Bland–Altman values based on the data plotted in Fig. 4

	Percentage of values within 95% confidence interval		
	RPM‐Anzai (%)	RPM‐Kinect (%)	Anzai‐Kinect (%)
Subject 1	96.09	96.44	98.93
Subject 2	96.03	93.38	94.04

**Figure 5:**
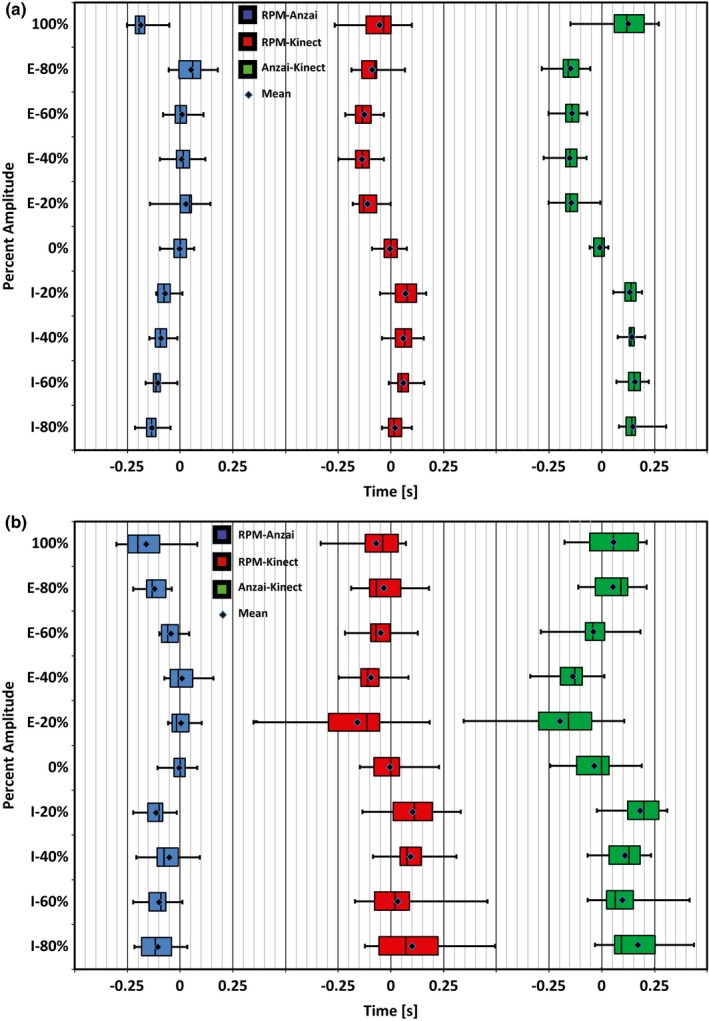
Box and Whiskers plots displaying the IQR of time differences obtained between products when utilizing an amplitude binning process. (a) Subject 1 and (b) Subject 2 both performed natural breathing patterns over a period of 120 s. “I” and “E” next to the percentage value on the *y*‐axis indicate “Inhalation” and “Exhalation”, respectively. The mean for each comparison is indicated with a point within each box.

**Figure 6:**
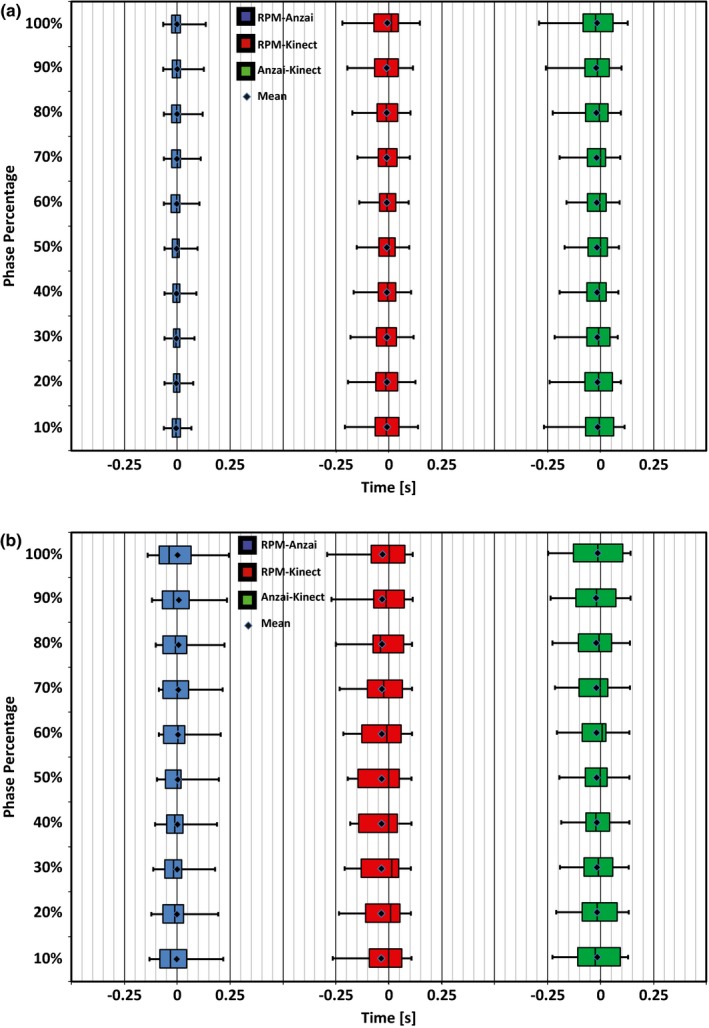
Box and Whiskers plots displaying the IQR of time differences obtained between products when utilizing a phase based binning process. (a) Subject 1 and (b) Subject 2 both performed natural breathing patterns over a period of 120 s. The mean for each comparison is indicated with a point within each box.

Lastly, the difference between the times obtained for each product within the amplitude and phase based binning process was calculated and the average difference across products for each percentage was calculated. Figure 5 displays the Interquartile Range (IQR) for the amplitude time differences by way of a Box and Whiskers plot for both subjects. Figure 6 displays the IQR for the phase time differences across each of the calculated bins utilizing similar Box and Whiskers plots for both subjects.

The IQR becomes an important quantifier when analyzing the differences between traces as it indicates a range of time that specific percentages of amplitude and phase differ between products. A summary of IQR values can be found in Tables 2 and 3 with Table 2 displaying the average time span within the IQR for each comparison and Table 3 containing the average mean time difference and standard deviation for each comparison.

**Table 2:** Average time spans of the Interquartile Range (Q3–Q1) calculated between each product with 2 subjects. Values were averaged over all 10 amplitude and 10 phase bins per cycle created in the above analysis

Binning process	Subject	Average IQR time spans [s]		
		RPM‐Anzai	RPM‐Kinect	Anzai‐Kinect
Amplitude	Subject 1	0.056	0.076	0.062
	Subject 2	0.095	0.157	0.160
Phase	Subject 1	0.036	0.096	0.110
	Subject 2	0.106	0.167	0.154

**Table 3:** Average time difference throughout trace between each product with 2 subjects. Values were averaged over all ten amplitude and ten phase bins per cycle created in the above analysis

Binning process	Subject	Average mean time difference throughout trace [s]		
		RPM‐Anzai	RPM‐Kinect	Anzai‐Kinect
Amplitude	Subject 1	0.009 ± 0.087	−0.002 ± 0.095	−0.011 ± 0.144
	Subject 2	0.020 ± 0.110	−0.007 ± 0.185	−0.026 ± 0.233
Phase	Subject 1	−0.137 ± 0.034	−0.031 ± 0.067	0.106 ± 0.0742
	Subject 2	−0.082 ± 0.086	−0.072 ± 0.115	0.010 ± 0.0984

When analyzing traces with the amplitude based binning process for each breathing cycle, the IQR for the time differences between products was low overall, typically lower than 0.2 s. Subject 1 had much better agreement across products with the IQR spanning a time frame of ~0.07 s, while the IQR for Subject 2 spanned a time frame of ~0.15 s.

The largest deviation when comparing all three products in this manner occurred for Subject 2 during the 100% portion (Max Inhalation) and 20% Exhalation portions of the curve. Here the IQR spanned ~0.25 s for both portions when comparing the Kinect to Anzai or RPM. However, when comparing RPM directly to Anzai, the 100% portion had a time span of ~0.12 s, whereas the 20% Exhalation bin spanned ~0.10 s.

When analyzing traces with the phase based binning process, the Kinect values from Subject 1 were, again, in much better agreement with RPM and Anzai belt compared with Subject 2, yet time differences for each bin between the products were still quite low. For Subject 1, the IQR spanned a time frame of ~0.08 s when comparing the Kinect to the RPM or Anzai verses a difference of ~0.07 s when RPM was compared to Anzai directly. For Subject 2, the IQR spanned a larger range of ~0.16 s when the Kinect was compared to RPM or Anzai but was ~0.12 s when RPM was compared directly to Anzai.

Given these ranges of time differences for the IQR, it becomes important to quantify how this would affect a 4DCT being generated by incorporating the couch feed. In our scanning protocols at Karmanos Cancer Institute, a typical 4DCT may include a couch pitch of 0.1, 0.5 gantry rotations/s, and detector configuration of 24 × 1.2 mm, giving the effective movement of the couch as 5.76 mm/s. Although the scans are helical in nature, we can estimate reconstruction differences of “effective slices” using this information and the variation between the respiratory traces. Assuming a constant rate of movement and 1.5 mm thick slices, it can be said that 3.84 effective slices are acquired every second with deviations of the expected time for slice acquisition creating a slice offset. Table 4 summarizes what minimal impact these IQR values would have during a 4DCT acquisition process.

**Table 4:** Summary of (a) couch movement and (b) fraction of slices that would have occurred during the IQR time spans calculated for each subject

(a)				
Binning process	Subject	Couch movement @ 5.76 mm/s [mm]		
		RPM‐Anzai	RPM‐Kinect	Anzai‐Kinect
Amplitude	Subject 1	0.35	0.42	0.37
	Subject 2	0.54	0.83	0.88
Phase	Subject 1	0.25	0.48	0.50
	Subject 2	0.67	1.01	1.05
(b)				
Binning process	Subject	Fraction of slice offset @ 3.84 slices/s		
		RPM‐Anzai	RPM‐Kinect	Anzai‐Kinect
Amplitude	Subject 1	0.17	0.21	0.18
	Subject 2	0.26	0.41	0.43
Phase	Subject 1	0.13	0.24	0.24
	Subject 2	0.33	0.50	0.52

## 4 DISCUSSION

The process of acquiring a respiratory trace utilizing the Kinect v2 sensor has shown the ability to provide results that are congruent to that of RPM and Anzai. Visually, when overlapping traces from all three products, there is minimal difference between them. When analyzing the traces through an amplitude and phase based binning process, time values associated with each amplitude and phase percentage were extracted and compared across each product. Using the Bland–Altman approach, it was shown that between 93% and 96% of the time values fell within the 95% confidence interval when comparing the Kinect to RPM and between 94% and 99% of the time values fell within the 95% confidence interval when comparing the Kinect to Anzai. These ranges indicate that each of the products recorded similar measurements to one another. Lastly, IRQ values were calculated for comparisons between products for the amplitude‐ and phase‐based binning processes. Again, values obtained for comparisons between the Kinect and RPM or Anzai were shown to be similar to those obtained when comparing RPM to Anzai. Deviations that did occur with the IRQ values in these comparisons were shown to have minimal effect on the couch movement or slice offsets that would occur during a 4DCT acquisition process.

One item of note is in regards to the time values obtained from the traces associated with Subject 2. The traces used in the analysis were noticeably more noisy than those used for Subject 1, indicating an overall reduction in the magnitude of the patient surface motion. When analyzing the raw data, it was found that the reduction in magnitude was evident in that the average difference between the maximum and minimum depth values of each respiratory cycle was 19.1 mm for Subject 1 but only 7.8 mm for Subject 2. As mentioned previously, the analysis performed for the Kinect traces was done so utilizing Point 5 (directly over the diaphragm). This point was chosen as the point of comparison simply to analyze a trace that would be obtained from a different location as the RPM and Anzai trace. Although Point 5 was shown to be accurate and comparable to both RPM and Anzai, increased agreement between products could be obtained if the system had automatically chosen the point based on the largest amplitude difference. With this criterion in mind, Point 9 (directly to the left of the RPM block) would have been chosen as the representation for respiratory motion. Here, the average difference between the maximum and minimum depth values for each respiratory cycle increased to 9.5 mm.

**Figure 7:**
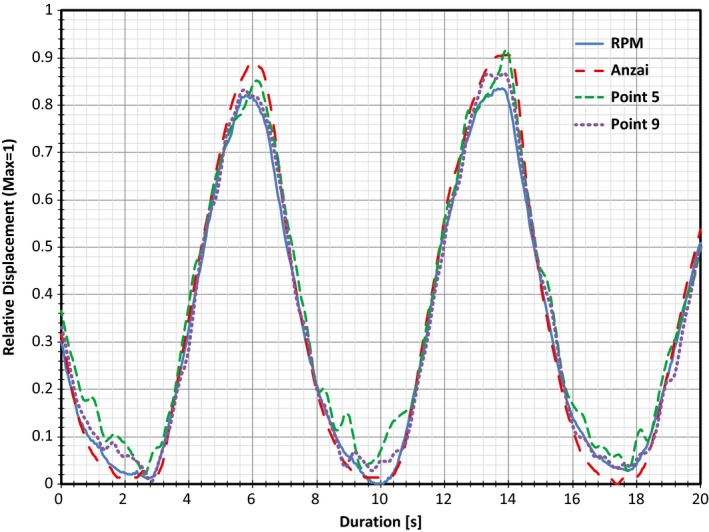
Overlapped respiratory traces obtained for Subject 2 utilizing RPM, Anzai, and both Point 5 (diaphragm) and Point 9 (left of RPM block) from the Kinect. Note the noise generated at maximum inhalation and exhalation for the trace associated with Point 5 has decreased significantly for the trace associated with Point 9.

The difference between the two points can be visualized in Fig. 7 which displays traces for RPM and Anzai overlapped with traces obtained for both Point 5 and Point 9 from the Kinect for Subject 2. Here, much of the noise present for Point 5 during maximum inhalation and maximum exhalation has dissipated for the trace associated with point 9. Additionally, Table 5 shows the change in average IQR time spans when comparing the traces from Point 5 and Point 9 to RPM and Anzai. It can be seen how the average IQR decreases with the trace from Point 9 to values that are closer to that of the RPM and Anzai comparison. This indicates that further study may be required to determine the effect that selections of different point on the body may have on noise introduced within the Kinect system.

**Table 5:** Comparison of the Average IQR time spans obtained for Subject 2's traces associated with Point 5 (diaphragm) and Point 9 (left of RPM block). Note the decrease in IQR time spans for Point 9 and their similarity to the RPM‐Anzai comparison

Binning process	Point	Subject 2 average IQR time spans [s]		
		RPM‐Anzai	RPM‐Kinect	Anzai‐Kinect
Amplitude	Point 5	0.095	0.157	0.160
	Point 9	0.095	0.098	0.108
Phase	Point 5	0.106	0.167	0.154
	Point 9	0.106	0.137	0.114

Although this analysis has shown the Kinect can produce similar traces to those of Anzai and RPM, the current iteration of respiratory tracking with the Kinect is not without its limitations. One issue encountered was in regards to the body tracking capabilities of the Kinect software. As the system was originally designed as a body‐tracking device for gaming, the optimal position for recognition and tracking is for the subject to be in a standing position, facing the camera directly. Given that a patient will be in a supine position on the CT couch, this can pose a problem. It was found that the body tracking system does perform well when turned on while a subject is moved onto the couch and into a lying position. However, when turned on while a subject is already lying on the couch, body tracking does not recognize the body as it cannot differentiate it from the couch. This failure of the system can be overcome by truncation of the color frame to match that of the depth frame. The patient will not be isolated on the screen with the background removed, but the selection of tracking points can continue in the same manner as before with a coordinate system shift between the color frame and depth frame. This will align the color pixel selected with a depth frame pixel in order to track the same data. Alternatively, third party body tracking programs have been created outside of the Kinect SDK that can be implemented with this code, should a more robust body tracking process be required by the end user for background removal.^24^

A second issue is with regard to gross patient motion during the tracking process. Without constant supervision of the image on the screen, the patient could move significantly and interrupt the respiratory tracking process. This can be overcome by implementing thresholds of maximum amplitude traced. For example, should a patient's typical breathing pattern involve a trough to peak amplitude value ~20 mm, setting a threshold of ±10 mm would then alert the user that gross motion has occurred**.** Secondary to this process, the depth frame can be utilized to track gross motion across the entire frame. By saving an initial state of the patient and continually comparing it to the current state, the depth values within the frame can be compared and analyzed to detect where in the frame motion has occurred. This is a process currently being investigated by this institution and can easily be implemented at the same time as respiratory tracking to ensure that the user would be alerted if gross motion were to occur.

## 5 CONCLUSION

Recording respiratory motion with the Kinect v2 by way of recording depth values for specific pixels on the depth image, rather than anatomical locations, has shown to be as accurate as the Varian RPM system and Anzai belt and is easily implemented. The ability to select multiple points on a patient to be used for respiratory tracking through the GUI, allows for a unique and user‐friendly setup. Without the need for a physical hardware attached to the patient for tracking, points can be selected anywhere on the patient, including the area of the tumor, without interfering with a CT scan or radiation therapy.

## CONFLICT OF INTEREST

The authors have no relevant conflicts of interest to disclose.
